# Lateral Sinus Thrombosis in Otology: a Review

**DOI:** 10.4084/MJHID.2010.027

**Published:** 2010-09-07

**Authors:** B. Viswanatha, Khaja Naseeruddin

**Affiliations:** 1 Professor of ENT, Victoria Hospital, Bangalore Medical College & Research Institute, Bangalore., INDIA; 2 Professor of ENT, Joint Director of Medical Education, Bangalore., INDIA

## Abstract

Lateral sinus thrombosis (LST) is usually occurs as a complication of middle ear infection. The involvement of lateral sinus during the course of ear infection was a well known complication in preantibiotic days. The decrease in the incidence of LST is due to the introduction of broad-spectrum antibiotics, early diagnosis and surgical treatment. Now, it is a rare complication of otitis media and poses a serious threat that warrants immediate medical and surgical treatment. The classical clinical picture is often changed by previous antibiotic therapy. An awareness of this rare potentially devastating condition and its varied presentations is necessary for early diagnosis and treatment. LST can also occur after head injury.

Anticoagulants are recommended in cases LST, where there is propagation of thrombus after surgery. The use of anticoagulants is not apart of standard care of patients with LST and was more common prior to the advent of antibiotics. Anticoagulants arrests the spread of thrombosis but may increase the risk of venous infarctions and should be used cautiously.

## Introducion:

The proximity of the middle ear and mastoid air cells to the dural venous sinuses predisposes them to thrombosis and thrombophlebitis secondary to infection and inflammation in the middle ear and mastoid.^1^

Lateral sinus thrombosis (LST) accounts for 6% of all intracranial complications in the era of antibiotic treatment of suppurative ear disease.^2^,^3^ It is generally considered the third or fourth most common complication among all intracranial complications of chronic otitis media.^4^ It is a rare well-known otogenic complication with serious consequences if left untreated.^5^ It is frequently associated with other intracranial and extracranial complications.^4^ The classical clinical picture is often changed by previous antibiotic therapy making the diagnosis of the occasional case of lateral sinus thrombosis more difficult by altering the expected clinical course.^6^–^8^ In addition, this complication may now be more difficult to diagnose on based clinical judgment because of associated complications and antibiotics may mask typical symptoms that can alert the physician to the diagnosis.^4^

The decreased incidence and changing clinical presentations due to previous antibiotics therapy have made the early diagnosis of LST more difficult for the physicians. Prompt diagnosis of LST requires high index of suspicion and firm understanding of the varied clinical presentations of LST.^9^

## Incidence:

LST is generally considered the third or fourth most common complication among all intracranial complications of chronic otitis media.^4^,^10^ It ranked second to meningitis in the pre antibiotic era as the most frequent fatal complication of otitis media and lateral sinus thrombosis occurred largely as a complication of acute otitis media.^11^ LST in the antibiotic era has been strongly associated with the adult age group and with chronic middle ear disease The incidence of LST has sharply declined since the advent of antibiotics.^12^

## Sex:

Many reports have documented a clear male predominance.^4^,^6^,^13^,^14^

## Pathophysiology:

The spread of otogenic infection to the lateral sinus may be through a coalescent or cholesteatomatous bone erosion or through a thrombophlebitic phenomenon.^4^,^15^,^16^

LST occurs when infection causes thrombophlebitis of small venules surrounding the outer dural wall of the sigmoid sinus, erosion of the bone overlying the sigmoid sinus such as with cholesteatoma, or infection that spreads to sigmoid sinus through a dehiscence in the overlying bone.^9^ It usually develops as a complication of chronic otitis media caused by the direct dissemination of the infection through the neighboring eroded bone.^17^ It is also reported in patient with an intact sigmoid plate indicating propagation by the thrombophlebitic spread through small emissary vein.^8^,^17^

Bony plate erosion by cholesteatoma, granulation tissue or coalescence initially causes perisinus abscess. Due to pressure of the perisinus abscess over the sinus wall, there will be pressure necrosis of the sinus wall and the intima, causing the adherence of fibrin, blood cells and platelets, resulting in the formation a mural thrombus. This mural thrombus gets infected and progresses to obliterating thrombus. Fresh thrombus is formed and may cause propagation proximally to the internal jugular vein, distally to other dural sinuses and through the mastoid emissary vein to the subcutis.Thrombus formation helps to localize the infection and it may be considered as a protective mechanism.^18^

## Clinical features:

A concise definition of clinical picture of LST secondary to chronic otitis media remains elusive because of variability in patient presentations, presence of concurrent complications, or preadmission treatments. Some patients have life threatening septicemia at diagnosis, where as in others, LST is relatively asymptomatic and detected only during imaging studies.^4^

Teichgraeber et al.^7^ extensively reviewed the publications on LST, both in the antibiotic and preantibiotic era. They found that LST was more common in adults and it was predisposed by chronic otitis media. Recent studies have shown that majority of LST cases are secondary to attico antral type of ear disease.^13^

Clinical features vary according to the stage of the disease. The most frequent presenting symptoms were headache, otalgia, fever, otorrhoea & vomiting and pain in the neck.^5^,^11^,^14^,^19^ Severe headache, otalgia, “picket-fence” fever, and papilloedema are regarded as specific symptoms and signs for LST.^4^,^6^,^7^,^20^,^21^ The classical case of lateral sinus thrombosis in pre antibiotic era typically produced a picket fence fever curve, due to periodic release of hemolytic streptococci from septic sinus thrombus^7^. With the advent of antibiotics, the classic picket fence pattern is less frequently seen, and the nature of the otogenic disease has shifted from acute to chronic otitis.^22^

Occlusion of the lumen of the sinus, interruption of cortical venous circulation results in headache, papilloedema and increased intracranial pressure. Tenderness and edema over mastoid (Griesinger’s sign) are pathagnomonic of lateral sinus thrombosis and reflex thrombosis of mastoid emissary vein.^11^ Extension of thrombophlebitis in to the jugular bulb and internal jugular vein may present as tender mass in the neck along or across stenocleidomastoid muscle. 9th, 10th, 11th cranial nerve may be paralyzed by the presence and pressure of clot in the jugular bulb.^23^ The clinical picture of LST may be unclear due to the masking effect of the previous use of antibiotics and the overlap of signs and symptoms with other concomitant infections.^18^

## Associated complications:

The presence of lateral sinus thrombosis mandates further investigation for additional intracranial complication. In preantibiotic era lateral sinus thrombosis had associated complication in 80% patient. The development of antibiotics has reduced the incidence of complication to 20%.^14^ Concurrent intracranial and extracranial complications are commonly encountered among patients with LST and include meningitis, intracranial abscesses, otitic hydrocephalus and internal jugular vein thrombosis.^21^,^22^,^24^–^27^

## Bacteriology:

Use of antibiotics have changed not only the clinical presentation of LST, but also bacteriology.^7^ Beta hemolytic streptococcus is no longer dominant organism.^7^,^15^ Since chronic, rather than acute infection more commonly precede lateral sinus thrombosis, cultures characteristically yields mixed flora including bacteroids, staphylococcus, enterobacteriaceae, proteus, pseudomonas and others species.^7^,^14^,^17^ Since antibiotics are commonly used during the prodromal ear infection, blood culture is often negative.^6^,^7^

## Radiology:

**Figure 1:**
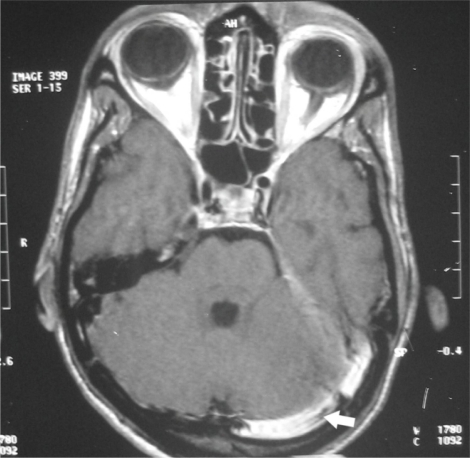
MRI scan showing (arrow) occlusion and dilatation of left transverse and lateral sinus (with permission from Indian journal of Otolaryngology & head and neck surgery).

Imaging in LST is considered a diagnostic aid, as definitive diagnosis of LST is made at surgery.^18^,^25^,^28^ CT and MRI are the investigations of choice in making diagnosis.^19^ CT scan is useful in demonstrating the classic ‘delta sign’ of perisinus dural enhancement and filling defect of the lateral sinus and also can help by ruling out other intracranial complications.^18^,^25^,^28^ MRI is more sensitive than CT in detecting the thrombus. It shows blood flow, sinus obstruction (Figure 1) and subsequent reversal of flow.^18^,^27^

**Figure 2:**
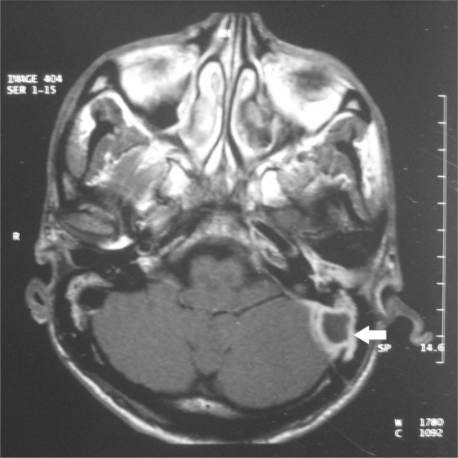
MRI scan showing (arrow) post contrast enhancement of the sinus wall on the left side (with permission from Indian journal of Otolaryngology & head and neck surgery).

MRI can show increased signal intensity of the thrombus and detect LST not identified on a routine CT.^23^,^28^ On gadolinium-enhanced MRI, thrombus appears as soft tissue signal associated with vascular bright appearance of the dural wall – the “delta” sign (Figure 2) as seen with gadolinium enhanced MRI.^19^,^28^ Additionally, MR venography, which can demonstrate the loss of signal and the absence of flow in the sinus, has proven to be more sensitive diagnostic tool in identifying LST.^23^,^28^ MRI is the investigation of choice, and should be performed in conjunction with CT, there by fully evaluating associated otologic and cerebral pathology.^28^ Magnetic resonance imaging is also useful for excluding an adjacent subdural empyema, cerebritis, or cerebral abscess.^29^

## Management:

Therapy of LST consists of administration of antibiotics and surgery.[18] Broad-spectrum intravenous antibiotics and removal of the infection source is the mainstay of therapy.^4^

In selected cases of LST, medical therapy alone with intravenous antibiotics may be successful. However, medical management requires prolonged administration of antibiotics.^3^,^14^ Conservative treatment of LST consisting of myringotomy (surgical incision on the tympanic membrane and pus drainage) and intravenous antibiotics without surgery is appropriate in highly selected cases secondary to acute otitis media in which prompt response to this treatment is seen.^29^

Lane performed the first successful surgery for lateral sinus thrombosis in 1888. Until then the mortality for this complication has been 100% (Goldenberg). Simple surgical management of LST in the pre antibiotic era reduced the mortality from virtually 100% to 30%.The addition of antibiotics further reduced the mortality to 10%.^3^,^21^,^30^

Mastoidectomy with incision of the lateral sinus, removal of the clot and local packing are considered standard care.^18^ Removal of all perisinus infection is mandatory for effective treatment.^4^ Free bleeding from both ends of the incised sinus is desirable. If there is no bleeding, evacuation of as much as clot as possible will suffice.^7^,^31^ Before the introduction of antibiotics it was felt desirable to obtain free bleeding from each ends of the incised sinus, but now it is thought be unnecessary to follow the clot centrally to obtain free bleeding nor it is thought be necessary to remove organized thrombus.^31^

In addition, the dilemma faced intraoperatively is the management of the thrombosed sinus. Conservative surgeons favor only needle aspiration of the sinus, citing the effectiveness of antibiotics.[4,14,15] Those who take a more aggressive approach prefer to remove the thrombus and to pack the sinus whether symptoms of septicemia are present or not.^4^,^10^,^18^,^21^ However recent, reports have challenged this dictum, and demonstrate that if the surrounding granulation tissue and inflammation are removed through a mastoidectomy, the sinus will reanalyze without clot evacuation.^1^

Seven et al.^4^ did not find any difference in outcome between patients who underwent only confirmatory needle aspiration and patients in whom the thrombus was removed. Many advocate incision of the sinus with removal of the clot, while other simply unroofs the sinus and confirm the presence of thrombus with needle aspiration.^22^

Recent reports have shown that if the surrounding granulation tissue and inflammation are removed through a mastoidectomy, the sinus will recannalize without clot evacuation. Jun et al 32 is of the opinion that the organized thrombus is an initial step for spontaneous resolution, finally inducing recanalizaiton of a sinus.

Anticoagulants therapy is not always indicated in the management of LST because; venous sinus has been shown to recannalize without anticoagulation following surgical evacuation of mastoid and epidural infection, and a 6-week course of antibiotics.^34^,^35^

Systemic anticoagulation has traditionally been achieved by initial intravenous treatment with unfractionated heparin, followed by transition to oral warfarin.^35^ It can be continued for three to four weeks. Anticoagulation limits propagation of thrombus and increases intracranial venous drainage and reduces the effects of raised intracranial pressure, resulting in improvement of neurological condition. Anticoagulation is useful in patients with multiple intracranial complications.^35^

Most authors agree that there is no place for anticoagulants in the management of LST.[7,18,20] The use of anticoagulants is not apart of standard care of patients with LST and was more common prior to the advent of antibiotics.^18^ Unless thrombus propagates after surgery, anticoagulants are not recommended.^14^,^33^ Anticoagulants arrests the spread of thrombosis but may increase the risk of venous infarctions and therefore no longer recommended.^19^

Although it is obvious that LST requires prompt mastoid surgeries with antibiotics for definitive management, the ligation of internal jugular vein is still a controversial issue in the management of LST. Internal jugular vein ligation was performed almost routinely in the pre antibiotic era, to avoid dissemination of thrombophlebitic process and septic emboli.^17^ In the modern antibiotic era, internal jugular vein ligation is reserved for those cases in which septicemia and embolization do not respond to initial surgery and antibiotic treatment.^11^,^19^,^31^,^36^

## Postoperative Details:

After the surgical treatment, the patient should be maintained on antibiotics for 2–3 weeks and a repeat MRI and MR venogram should be performed to rule out the development of secondary intracranial complication such as brain abscess, or propagation of thrombus in to the superior sagital sinus.^1^ Cases of secondary intracranial complication have been reported in the literature.^37^

## Morbidity:

Syms et al 14 reported an incidence of approximately 30% morbidity associated with otogenic LST. These include septic cardiomyopathy, acute respiratory distress syndrome, seizure, ventriculoperitoneal shunt, and upper extremity weakness.^4^

## Mortality:

In the preantibiotic era, mortality after otogenic LST was nearly 100%. In the antibiotic era, this has dropped to 0% to 25%. This is due to availability of broad-spectrum antibiotics and improved diagnostic tools.^4^,^7^,^10^,^14^ In recent years (Table 1) there have been less reports of OLST-related mortality.^5^,^13^,^22^,^33^

## Conclusion:

Lateral sinus thrombosis is now a rare intracranial complication of otitis media. An awareness of this condition is necessary for early diagnosis and treatment. It frequently occurs in association with other intracranial complications. Computed tomography and magnetic resonance imaging plays an important role in the management of this disease. Aggressive medical therapy with broad-spectrum intravenous antibiotics and removal of source of infection by surgery are required for a good outcome.

## Figures and Tables

**Table 1:** Showing studies by the authors

**Year**	**Authors name**	**Study period (yrs)**	**Cases**	**Mortality (%)**
1982	Teichgraber et al^7^	10	6	16%
1987	Samuel et al^21^	6	45	0%
1988	Amiramjdi^6^	7	16	0%
1990	O’Connell^8^	-	3	0%
1999	Sym’s et al^14^	5	6	0%
2002	Bardley et al^33^	6	9	0%
2003	Ooi et al^5^	4	4	0%
2006	B.Viswanatha^13^	7	12	0%
2009	Nathan^22^	11	7	0%
